# Impact of right atrial enlargement on clinical outcome in patients with atrial fibrillation

**DOI:** 10.3389/fcvm.2022.989012

**Published:** 2022-09-23

**Authors:** Kyu-Yong Ko, Ji-Hun Jang, Seong-Huan Choi, Yong-Soo Baek, Sung Woo Kwon, Sang-Don Park, Seong-Ill Woo, Dae-Hyeok Kim, Sung-Hee Shin

**Affiliations:** Division of Cardiology, Department of Internal Medicine, Inha University College of Medicine, Incheon, South Korea

**Keywords:** atrial fibrillation, left atrium, right atrium, heart failure, stroke

## Abstract

**Background:**

Left atrial (LA) remodeling is associated with adverse cardiovascular events, including heart failure (HF) and stroke in patients with atrial fibrillation (AF). However, there are limited data on the value of right atrial (RA) remodeling in this population. We investigated the prognostic role of RA enlargement in patients with non-valvular AF.

**Methods and results:**

We analyzed 254 consecutive patients (age = 69 ± 12years, male:female = 165:89, mean left ventricular ejection fraction = 58.0 ± 7.2%) with non-valvular AF who underwent two-dimensional echocardiography from a single center. RA and LA volumes were measured from apical views and indexed to the body surface areas (right atrial volume index [RAVI] and left atrial volume index [LAVI]) and RAVI > 30mL/m^2^ and LAVI > 34mL/m^2^ were considered as enlarged. The relationship between RA enlargement and composite clinical outcome of hospitalization for HF (HHF), stroke, systemic embolism, or death from any cause was assessed. Right atrial (RA) enlargement was associated with older age and more frequent prevalence of persistent or permanent AF. During a median follow-up of 47.1 months, 77 patients (30%) had experienced primary composite outcome. In a multivariable model, RA enlargement, but not LA enlargement, was independently associated with adverse clinical outcomes even after adjusting for clinical and echocardiographic factors {adjusted hazard ratio [HR], 1.90 [95% confidence interval (CI), 1.14–3.18], *p* = 0.014 for primary composite outcome; adjusted HR, 2.70 [95% CI, 1.27–5.67], *p* = 0.001 for HHF or all cause death}.

**Conclusion:**

RA enlargement was independently associated with an increased risk of HF, stroke, systemic embolization or death in patients with non-valvular AF, suggesting that RA volume can be helpful in assessing future cardiovascular risk in this population.

## Introduction

Atrial fibrillation (AF) is one of the most common arrhythmias. It affects approximately 6% of the population older than 65 years, and it is related to increased cardiovascular (CV) mortality and morbidity including heart failure (HF) and stroke (1–3).

It is common to have an enlarged left atrial (LA) size in patients with AF. While AF can increase LA size, LA structural change can also be related to AF development or recurrence after cardioversion or catheter ablation (4–6). Furthermore, impaired LA contraction in AF can result in stasis of blood and increase thromboembolic risk including stroke (7–9). The importance of LA remodeling has been well known in patients with various CV diseases (10–12). Although several studies have previously demonstrated that increased RA volume is a predictor of poor prognosis and low functional status in patients with HF, data are still limited on the value of RA remodeling in patients with AF (13, 14). In this study, we assessed whether RA volume was related to clinical outcomes in patients with non-valvular AF.

## Materials and methods

### Study population

We retrospectively enrolled 582 consecutive patients with AF who performed two-dimensional (2D)-echocardiography from May 2016 to February 2018 in our single institution and were followed up regularly. Patients were excluded if they had significant intrinsic valvular heart disease which is more than moderate degree on echocardiography, chronic obstructive pulmonary disease, malignancy, cardiac tumors, pheochromocytoma, severe renal failure requiring dialysis, cardiomyopathies, congenital cardiac abnormalities, rhythm disturbance requiring permanent pacemakers, left ventricular (LV) ejection fraction (EF) less than 45%, regional wall motion abnormalities, a poor acoustic window for adequate RA and LA assessment by echocardiography, or lost to follow-up.

AF was diagnosed by 12-lead electrocardiography (ECG) or 24-hour Holter monitoring. Paroxysmal AF and persistent AF were defined as an episode of self-terminating AF that lasts less than 7 days and longer than 7 days, respectively. Permanent AF is defined as AF that has been accepted by patients and physicians and has no further attempts to restore or maintain sinus rhythm (15–17).

**FIGURE 1:**
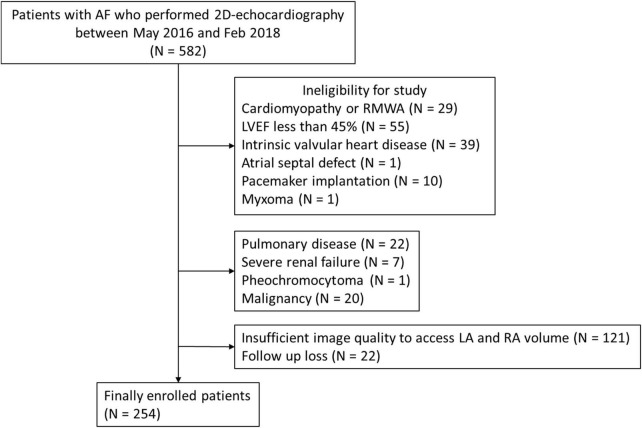
Study flow chart. AF, atrial fibrillation; LA, left atrium; LVEF, left ventricular ejection fraction; RA, right atrium; RWMA, regional wall motion abnormality.

In initially enrolled patients, 135 patients were excluded because of cardiomyopathies, LVEF <45%, intrinsic valvular disease, congenital heart disease, implanted pacemaker, or cardiac tumor. An additional 50 subjects were excluded due to pulmonary disease, severe renal failure, pheochromocytoma or malignancy. Among the remaining 397 patients, there was insufficient image quality for RA volume measurement, including substantial foreshortening in 121 patients and 22 patients had missing clinical data. Finally, 254 patients (mean age 69 ± 12 years, male:female = 165:89, mean LVEF = 58.0 ± 7.2%) were included in our analysis (Figure 1).

This study design was approved by the Institutional Review Board (INHAUH2022-01-034-000) and was conducted in compliance with the ethical principles outlined in the Declaration of Helsinki.

### Echocardiographic measurements

Standard echocardiographic parameters were measured in accordance with the American Society of Echocardiography guidelines (18). Standard 2D, M-mode, and Doppler images were acquired from parasternal, apical, and subcostal views. The measurements were averaged over 5 cardiac cycles. LV end-diastolic volume (LVEDV) and LV end-systolic volume (LVESV) were measured and LVEF was calculated using the modified Simpson’s method from apical four-chamber and two-chamber views. LV mass was calculated using the linear method from M-mode images. LA volume was measured from apical four and two-chamber views and RA volume was measured in apical four-chamber view at the end of LV systole by using the disks summation technique. LV mass, LA volume, and RA volume were indexed for body size by dividing by the body surface area. RA volume index (RAVI) > 30mL/m^2^ and LA volume index (LAVI) > 34mL/m^2^ were considered as enlarged, based on the European Association of Cardiovascular Imaging guideline (19). Early mitral inflow (E) velocity, deceleration time of the E wave, and septal mitral annular velocity (e’) were obtained from tissue Doppler imaging. Systolic pulmonary arterial pressure (SPAP) was calculated from the tricuspid regurgitation (TR) jet velocity and estimated RA pressure.

### Clinical data and follow-up

Clinical factors including age, gender, height, weight, history of HF, hypertension, diabetes mellitus, cerebral vascular events, rhythm control strategy, and type of AF were assessed. The patients were followed up regularly every 1–3 months, sometimes every 6 months, as determined by their physicians with a median follow-up of 47.1 [18.3–57.8] months. We evaluated the relationship of RA enlargement to clinical outcome. Primary clinical outcome included composite of hospitalization for HF (HHF), stroke, systemic embolism, or death from any causes. We additionally assessed the association between RA enlargement and a composite of HHF or all cause death. HHF was defined as the presentation of typical signs and symptoms requiring intravenous diuretics use and overnight hospitalization. Stroke was defined as sudden onset of neurologic deficit with new cerebral infarction on the brain magnetic resonance imaging. Systemic embolism was defined as acute vascular occlusion of an extremity or organ as documented by computed tomography.

### Statistical analysis

Continuous variables are described as mean ± standard deviation or median with interquartile range, as appropriate. Categorical variables are reported as number and percentages. Student *t*-test or Mann–Whitney U test was used for continuous variables, and the Chi-squared test or Fisher’s exact test was used for categorical variables to compare the baseline characteristics between the groups. The Kaplan-Meier analysis and log-rank test were used to compare event-free survival rates between the two groups, which were divided based on the RAVI. Cox proportional hazard model was additionally performed to assess the relationship between RA enlargement and clinical outcome. The proportional hazards assumption was tested for all analyses. The multivariable models adjusted for demographic and clinical covariates including age, gender, body mass index (BMI), diabetes, hypertension, type of AF, LA enlargement and LVEF, which were significantly related to clinical outcome or clinically relevant. To avoid overfitting, additional adjustment was made for LV mass index (LVMI), E/e’, SPAP and TR. We used spline regression model to assess the continuous relationship of RAVI with clinical outcomes. To clarify the effect of RA enlargement on clinical events independent of LA enlargement, we additionally performed a sensitivity analysis for the relationship between RA enlargement and clinical outcomes in patients with LA enlargement.

For all analyses, a 2-sided *p* ≤ 0.05 was considered statistically significant. R statistical software (version 4.1.0; R Foundation for Statistical Computing, Vienna, Austria) was used for the analysis.

## Results

### Baseline clinical and echocardiographic characteristics

Table 1 showed the baseline characteristics and echocardiographic parameters of the study population with and without RA enlargement. Patients with enlarged RA were older (72 ± 11 years vs. 67 ± 13 years, *p* < 0.001), had lower BMI (23.7 ± 3.3kg/m^2^ vs. 25.1 ± 3.2kg/m^2^, *p* < 0.001), and had persistent or permanent AF more frequently (89.5% vs. 57.9%, *p* < 0.001) than those without RA enlargement. In addition, RA enlargement was associated with greater LV mass and LA volume, higher SPAP, and more significant TR (108.1 ± 21.9 g/m^2^ vs. 100.6 ± 22.3 g/m^2^, *p* = 0.002 for LVMI; 69.1 ± 23.2 mL/m^2^ vs. 44.7 ± 18.9 mL/m^2^, *p* < 0.001 for LAVI; 35.3 ± 11.4 mmHg vs. 29.5 ± 7.2 mmHg, *p* < 0.001 for SPAP; 17.3% vs. 2.8%, *p* = 0.006 for moderate or severe TR). Prescription rate of anticoagulants tended to be higher in patients with RA enlargement, but it was not statistically significant. The proportion of patients with a history of HF hospitalization was only 3.5%, which was comparable between the groups.

**TABLE 1:** Baseline characteristics of the study population according to the right atrial (RA) size.

	RAVI > 30mL/m^2^ (*n* = 114)	RAVI ≤ 30mL/m^2^ (*n* = 140)	Total (*n* = 254)	*P*-value
**Clinical characteristics**				
Age, years	72 ± 11	67 ± 13	69 ± 12	<0.001*
Female, n (%)	40 (35.1%)	49 (35.0%)	89 (35.0%)	>0.999
BMI (kg/m^2^)	23.7 ± 3.3	25.1 ± 3.2	24.5 ± 3.3	0.001*
Prior history of HF, n (%)	5 (4.4%)	4 (2.9%)	9 (3.5%)	0.520
Prior history of Stroke, n (%)	16 (14.0%)	11 (7.9%)	27 (10.6%)	0.166
Hypertension, n (%)	68 (59.6%)	80 (57.1%)	149 (58.3%)	0.783
Diabetes mellitus, n (%)	25 (21.9%)	31 (22.1%)	56 (22.0%)	>0.999
Type of AF, n (%)				<0.001*
Paroxysmal	12 (10.5%)	59 (42.1%)	71 (23.0%)	
Persistent or permanent	102 (89.5%)	81 (57.9%)	183 (72.0%)	
Oral anticoagulants, n (%)	39 (34.2%)	33 (23.6%)	72 (28.3%)	0.083
Rhythm control, n (%)	11 (9.6%)	21 (15.0%)	32 (12.6%)	0.277
AF ablation	3 (2.6%)	14 (10.0%)	17 (6.7%)	0.037*
Antiarrhythmic drugs	9 (7.9%)	11 (7.9%)	20 (7.9%)	>0.999
**Echocardiographic parameters**				
LVEDV (mL)	76.5 ± 23.0	71.6 ± 20.2	73.8 ± 21.6	0.149
LVESV (mL)	32.9 ± 13.0	29.9 ± 10.5	31.3 ± 11.8	0.094
LVMI (g/m^2^)	108.1 ± 21.9	100.6 ± 22.3	103.9 ± 22.4	0.002*
LVEF (%)	57.5 ± 7.8	58.4 ± 6.6	58.0 ± 7.2	0.275
LAVI (mL/m^2^)	69.1 ± 23.2	44.7 ± 18.9	57.5 ± 23.5	<0.001*
RAVI (mL/m^2^)	45.7 ± 14.2	22.1 ± 5.1	32.7 ± 15.6	<0.001*
LA enlargement, n (%)	113 (99.1%)	117 (83.6%)	230 (90.6%)	<0.001*
E(cm/s)	88.7 ± 18.7	83.1 ± 20.5	85.6 ± 19.8	0.016*
e’ (cm/s)	7.4 ± 1.9	7.7 ± 2.2	7.6 ± 2.1	0.194
E/e’	12.7 ± 4.2	11.6 ± 4.7	12.1 ± 4.5	0.010*
DT (msec)	170.0 ± 32.6	184.4 ± 41.4	177.9 ± 38.3	0.022*
SPAP (mmHg)	35.3 ± 11.4	29.5 ± 7.2	32.2 ± 9.8	<0.001*
TR Vmax (m/s)	2.6 ± 0.4	2.4 ± 0.3	2.5 ± 0.4	<0.001*
MR grade, n (%)				<0.001*
No	36 (31.6%)	94 (67.1%)	130 (51.2%)	
Mild	73 (64.0%)	45 (32.1%)	118 (46.5%)	
Moderate/severe	5 (4.4%)	1 (0.8%)	6 (2.3%)	
TR grade, n (%)				<0.001*
No	16 (14.0%)	68 (48.6%)	84 (33.1%)	
Mild	81 (71.1%)	70 (50.0%)	151 (59.4%)	
Moderate/severe	17 (14.9%)	2 (1.4%)	19 (7.5%)	

Variables are mean ± SD, n (%). AF, atrial fibrillation; BMI, body mass index; DT, deceleration time; HF, heart failure; LA, left atrium; LAVI, left atrial volume index; LVEDV, left ventricular end diastolic volume; LVEF, left ventricular ejection fraction; LVESV, left ventricular end systolic volume; LVMI, left ventricular mass index; MR, mitral regurgitation; RAVI, right atrial volume index; SPAP, systolic pulmonary atrial pressure; TR, tricuspid regurgitation. * Mean *p* < 0.05.

### Association between right atrial size and clinical events

During a median follow-up of 47.1 months, 77 patients (30%) experienced primary composite endpoint. 37 patients (15%) were hospitalized for HF, 41 patients (16%) were hospitalized for stroke, 6 patients (2%) had systemic embolization, and death occurred in 6 patients (2%).

**FIGURE 2:**
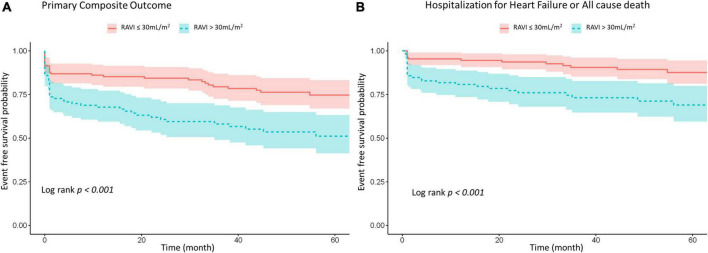
Kaplan–Meier survival analysis for **(A)** primary composite outcome of HHF, stroke, systemic embolism or all cause death and **(B)** composite of HHF or all cause death according to RA size. HHF, hospitalization for heart failure; RA, right atrium; RAVI, right atrial volume index.

**FIGURE 3:**
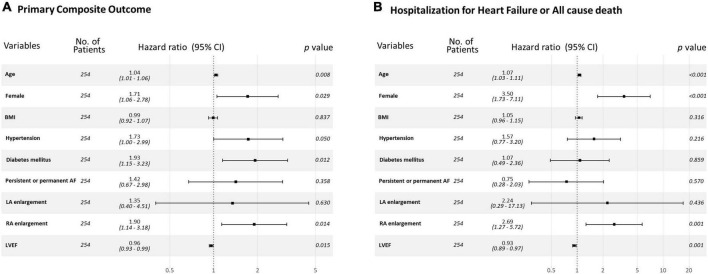
Forest plot for **(A)** primary composite outcome and **(B)** a composite of HHF or all cause death for each variable. HHF, hospitalization for heart failure.

Kaplan-Meier curve showed that the higher estimated event rate in patients with RA enlargement than those without RA enlargement (Figure 2, Log rank *p* < 0.001). In univariate Cox proportional hazard analysis, age, gender, BMI, hypertension, diabetes, type of AF, and rhythm control strategy were associated with composite outcome (Table 2). Regarding echocardiographic parameters, RA enlargement, LVMI, E/e’, pulmonary hypertension, and moderate or severe TR were related to clinical events {Table 2, Hazard Ratio (HR), 2.34 [95% Confidence interval (CI), 1.48–3.70], *p* < 0.001 for primary composite outcome of RA enlargement; HR, 2.75 [95% CI, 1.44–5.24], p = 0.002 for HHF or all cause death of RA enlargement}. After adjusting for clinical and echocardiographic parameters, RA enlargement and LVEF were independently associated with adverse clinical events (Figure 3 and Tables 3, 4, and, adjusted HR, 1.90 [95% CI, 1.14–3.18], *p* = 0.014 for primary composite outcome of RA enlargement; adjusted HR, 2.70 [95% CI, 1.27–5.67], *p* = 0.001 for HHF or all cause death of RA enlargement). The association of RA enlargement with primary composite outcome remained significant after additional adjusting for LVMI, E/e’, pulmonary hypertension, or TR. In contrast, LA enlargement was not significantly associated with adverse events.

**TABLE 2:** Univariate Cox proportional hazard analysis for primary composite outcomes of HHF, stroke, systemic embolism or all cause death, and composite end-point of HHF or all cause death.

Variables	Primary composite outcome (*n* = 77)		HHF or all cause death (*n* = 41)	
	HR (95% CI)	*P*-value	HR (95% CI)	*P*-value
**Clinical characteristics**				
Age	1.06 (1.03–1.08)	<0.001*	1.09 (1.05–1.12)	< 0.001*
Female	2.15 (1.37–3.37)	<0.001*	4.53 (2.34–8.76)	< 0.001*
BMI	0.92 (0.86–0.99)	0.020*	0.91 (0.83–1.00)	0.060
Prior history of HF	1.99 (0.81–4.94)	0.136	3.76 (1.34–10.56)	0.012*
Prior history of Stroke	1.69 (0.91–3.13)	0.094	1.19 (0.47–3.02)	0.721
Hypertension	2.35 (1.41–3.91)	0.001*	1.91 (0.98–3.75)	0.059
Diabetes mellitus	2.06 (1.28–3.30)	0.003*	0.99 (0.47–2.08)	0.981
Persistent or permanent AF	2.99 (1.54–5.81)	0.001*	2.38 (1.00–5.67)	0.049*
Oral anticoagulants	0.87 (0.52–1.45)	0.590	0.96 (0.48–1.93)	0.918
Rhythm control	0.23 (0.07–0.73)	0.013*	0.49 (0.15–1.59)	0.234
AF ablation	0.15 (0.02–1.05)	0.056	0.30 (0.04–2.18)	0.233
Antiarrhythmic drugs	0.26 (0.07–1.07)	0.062	0.55 (1.13–2.27)	0.408
**Echocardiographic parameters**				
LA enlargement (≥ 34mL/m^2^)	2.93 (0.92–9.29)	0.068	4.80 (0.66–34.89)	0.121
RA enlargement (≥ 30mL/m^2^)	2.34 (1.48–3.70)	<0.001*	2.75 (1.44–5.24)	0.002*
LAVI (mL/m^2^)	1.02 (1.01–1.02)	<0.001*	1.02 (1.01–1.03)	< 0.001*
RAVI (mL/m^2^)	1.03 (1.01–1.04)	<0.001*	1.04 (1.02–1.05)	< 0.001*
LVEDV (mL)	0.99 (0.98–1.00)	0.127	0.98 (0.967–1.00)	0.020*
LVESV (mL)	1.00 (0.98–1.02)	0.619	0.99 (0.96–1.02)	0.500
LVMI (g/m^2^)	1.02 (1.01–1.02)	<0.001*	1.02 (1.00–1.03)	0.008*
LVEF (%)	0.98 (0.95–1.01)	0.181	0.95 (0.91–0.99)	0.017*
E/e’	1.10 (1.07–1.14)	<0.001*	1.12 (1.07–1.17)	< 0.001*
DT(msec)	0.99 (0.99–1.00)	0.010*	0.99 (0.98–1.00)	0.018*
SPAP (mmHg)	1.03 (1.01–1.05)	0.001*	1.04 (1.02–1.06)	< 0.001*
MR	2.64 (0.96–7.24)	0.059	2.91 (0.70–12.08)	0.141
TR	2.45 (1.29–4.64)	0.006*	3.86 (1.78–8.37)	< 0.001*

AF, atrial fibrillation; BMI, body mass index; CI, confidence interval; DT, deceleration time; HF, heart failure; HHF, heart failure hospitalization; HR, hazard ratio; LA, left atrium; LVEDV, left ventricular end diastolic volume; LVEF, left ventricular ejection fraction; LVESV, left ventricular end systolic volume; LVMI, left ventricular mass index; MR, mitral regurgitation; RA, right atrium; SPAP, systolic pulmonary atrial pressure; TR, tricuspid regurgitation. * Mean *p* < 0.05.

**TABLE 3:** Multivariate Cox proportional hazard analysis for primary composite outcomes of HHF, stroke, systemic embolism or all cause death.

Variables	Primary composite outcome (*n* = 77)									
	Model 1^†^		Model 1 + LVMI		Model 1 + E/e’		Model 1 + SPAP		Model 1 + TR	
	HR (95% CI)	*P*-value	HR (95% CI)	*P*-value	HR (95% CI)	*P*-value	HR (95% CI)	*P*-value	HR (95% CI)	*P*-value
**Clinical characteristics**										
Age	1.04 (1.01–1.06)	0.008*	1.03 (1.01–1.06)	0.016*	1.02 (1.00–1.05)	0.111	1.04 (1.01–1.07)	0.012*	1.03 (1.01–1.06)	0.011*
Female	1.72 (1.06–2.78)	0.029	1.72 (1.06–2.79)	0.028*	1.47 (0.88–2.44)	0.140	1.59 (0.97–2.59)	0.065	1.70 (1.05–2.76)	0.031*
BMI	0.99 (0.92–1.07)	0.837	0.99 (0.92–1.07)	0.853	0.99 (0.92–1.07)	0.818	1.00 (0.93–1.07)	0.945	0.99 (0.92–1.07)	0.797
Hypertension	1.73 (1.00–3.00)	0.050	1.65 (0.95–2.87)	0.077	1.84 (1.05–3.20)	0.032*	1.86 (1.06–3.26)	0.030*	1.78 (1.04–3.08)	0.037*
Diabetes mellitus	1.93 (1.15–3.23)	0.012*	1.95 (1.16–3.26)	0.012*	1.73 (1.02–2.94)	0.041*	1.95 (1.16–3.26)	0.011*	1.97 (1.18–3.30)	0.010*
Persistent or permanent AF	1.42 (0.67–2.98)	0.358	1.40 (0.67–2.94)	0.373	1.47 (0.70–3.10)	0.315	1.27 (0.61–2.68)	0.525	1.42 (0.67–2.98)	0.359
**Echocardiographic parameters**										
LA enlargement (≥ 34mL/m^2^)	1.35 (0.40–4.52)	0.630	1.42 (0.42–4.77)	0.573	1.16 (0.34–3.94)	0.809	1.29 (0.38–4.34)	0.686	1.31 (0.39–4.41)	0.661
RA enlargement (≥ 30mL/m^2^)	1.90 (1.14–3.18)	0.014*	1.88 (1.12–3.16)	0.017*	2.01 (1.20–3.67)	0.008*	1.86 (1.08–3.21)	0.026*	1.83 (1.09–3.07)	0.023*
LVEF (%)	0.96 (0.93–0.99)	0.015*	0.97 (0.94–1.00)	0.042*	0.97 (0.94–1.00)	0.025*	0.96 (0.93–0.99)	0.009*	0.96 (0.93–0.99)	0.013*
LVMI (g/m^2^)	–	–	1.01 (1.00–1.02)	0.204	–	–	–	–	–	–
E/e’	–	–	–	–	1.06 (1.01–1.11)	0.014*	–	–	–	–
SPAP (mmHg)	–	–	–	–	–	–	1.01 (0.99–1.03)	0.394	–	–
TR	–	–	–	–	–	–	–	–	1.58 (0.81–3.06)	0.178

^†^Model 1 included age, sex, BMI, hypertension, diabetes, type of AF, LA enlargement, RA enlargement and LVEF. AF, atrial fibrillation; BMI, body mass index; CI, confidence interval; HR, hazard ratio; LA, left atrium; LVMI, left ventricular mass index; RA, right atrium; SPAP, systolic pulmonary atrial pressure; TR, tricuspid regurgitation * Mean *p* < 0.05.

**TABLE 4:** Multivariate Cox proportional hazard analysis for composite end–point of HHF or all cause death.

Variables	HHF or all cause death (*n* = 41)									
	Model 1^†^		Model 1 + LVMI		Model 1 + E/e’		Model 1 + SPAP		Model 1 + TR	
	HR (95% CI)	*P*-value	HR (95% CI)	*P*-value	HR (95% CI)	*P*-value	HR (95% CI)	*P*-value	HR (95% CI)	*P*-value
**Clinical characteristics**										
Age	1.07 (1.03–1.11)	< 0.001*	1.07 (1.03–1.11)	< 0.001*	1.06 (1.02–1.10)	0.003*	1.07 (1.03–1.11)	< 0.001*	1.07 (1.03–1.11)	< 0.001*
Female	3.50 (1.73–7.11)	< 0.001*	3.52 (1.73–7.14)	< 0.001*	3.11 (1.50–6.43)	0.002*	2.99 (1.46–6.12)	0.003*	3.44 (1.70–6.67)	< 0.001*
BMI	1.05 (0.96–1.15)	0.316	1.05 (0.96–1.15)	0.320	1.07 (0.97–1.17)	0.188	1.05 (0.96–1.16)	0.271	1.05 (0.96–1.15)	0.308
Hypertension	1.57 (0.77–3.20)	0.216	1.53 (0.74–3.19)	0.252	1.57 (0.77–3.18)	0.214	1.81 (0.87–3.74)	0.112	1.70 (0.84–3.42)	0.140
Diabetes mellitus	1.07 (0.49–2.36)	0.859	1.07 (0.49–2.36)	0.865	0.94 (0.42–2.13)	0.890	1.01 (0.46–2.23)	0.978	1.12 (0.51–2.44)	0.784
Persistent or permanent AF	0.75 (0.28–2.03)	0.570	0.75 (0.28–2.04)	0.577	0.74 (0.27–2.03)	0.563	0.64 (0.23–1.75)	0.381	0.78 (0.29–2.09)	0.618
**Echocardiographic parameters**										
LA enlargement (≥ 34mL/m^2^)	2.24 (0.29–17.13)	0.436	2.27 (0.30–17.42)	0.430	1.89 (0.24–14.71)	0.542	2.51 (0.32–19.52)	0.380	2.14 (0.28–16.33)	0.465
RA enlargement (≥ 30mL/m^2^)	2.70 (1.27–5.67)	0.001*	2.65 (1.23–5.69)	0.013*	3.02 (1.39–6.54)	0.005*	2.44 (1.05–5.65)	0.037*	2.32 (1.07–4.93)	0.029*
LVEF (%)	0.93 (0.89–0.97)	0.001*	0.93 (0.89–0.97)	0.002*	0.93 (0.89–0.98)	0.003*	0.91 (0.87–0.96)	< 0.001*	0.92 (0.87–0.96)	< 0.001*
LVMI (g/m^2^)	–	–	1.00 (0.99–1.02)	0.785	–	–	–	–	–	–
E/e’	–	–	–	–	1.05 (0.99–1.12)	0.131	–	–	–	–
SPAP (mmHg)	–	–	–	–	–	–	1.03 (1.00–1.06)	0.061	–	–
TR	–	–	–	–	–	–	–	–	2.80 (1.21–6.48)	0.016*

^†^Model 1 included age, sex, BMI, hypertension, diabetes, type of AF, LA enlargement, RA enlargement and LVEF. AF, atrial fibrillation; BMI, body mass index; CI, confidence interval; HHF, heart failure hospitalization; HR, hazard ratio; LA, left atrium; LVMI, left ventricular mass index; RA, right atrium; SPAP, systolic pulmonary atrial pressure; TR, tricuspid regurgitation * Mean *p* < 0.05.

Rhythm control strategy was not significantly associated with adverse events in multivariable analysis (*p* = 0.12 for primary composite outcome, *p* = 0.77 for HHF or all cause death). When adding the rhythm control to the standard model in a multivariable model, RA enlargement was consistently associated with adverse clinical outcomes (adjusted HR, 1.95 [95% CI, 1.16–3.25], *p* = 0.011 for primary composite outcome; adjusted HR, 2.68 [95% CI, 1.27–5.69], *p* = 0.010 for HHF or all cause death).

**FIGURE 4:**
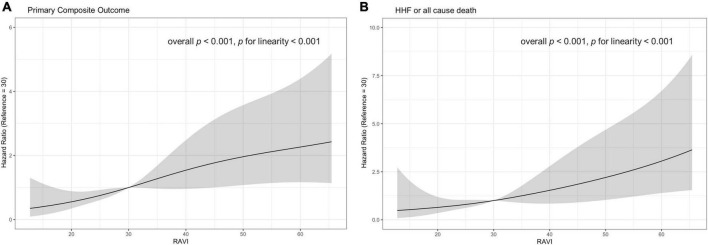
Estimated hazard ratio with RAVI and **(A)** primary composite outcome **(B)** a composite outcome of HHF or all cause death. The solid curves indicating unadjusted estimates and colored curves indicating 95% confidence limits. HHF, hospitalization for heart failure; RAVI, right atrial volume index.

When assessing RA volume as continuous variables, RAVI showed a linear association with the clinical outcome (Figure 4). Higher value of RAVI was associated with a higher risk of both primary composite outcome and a composite of HHF or all cause death in multivariable model (adjusted HR, 1.02 [95% CI, 1.00–1.03], *p* = 0.007 for primary composite outcome per 1mL/m^2^; adjusted HR, 1.04 [95% CI, 1.02–1.06], *p* < 0.001 for HHF or all cause death per 1mL/m^2^) whereas LAVI was not associated clinical outcomes.

**FIGURE 5:**
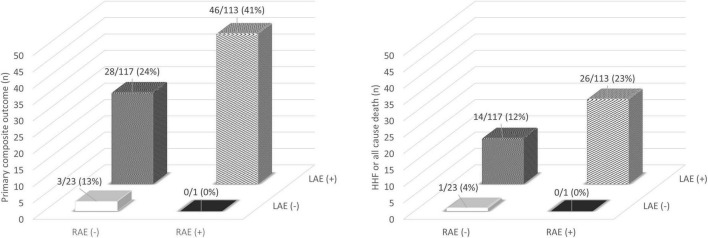
Incidence of adverse clinical outcome classified by atrial enlargement. HHF, hospitalization for heart failure; LAE, left atrial enlargement; RAE, right atrial enlargement.

When the patients were divided by RA and LA enlargement, patients with RA enlargement had adverse events more frequently than in those without RA enlargement among patients with LA enlargement (Figure 5). The event rates were approximately 1.7-fold higher for primary composite outcome and 1.9-fold higher for HFF or all cause death in the group with RA enlargement when compared with those without RA enlargement among the patients with LA enlargement.

## Discussion

In our study, RA enlargement was related to older age, female gender, lower body weight, and longer duration of AF in patients with non-valvular AF. In addition, RA size was associated with LVMI, elevated E/e’, SPAP and tricuspid regurgitation. After adjusting for clinical and echocardiographic factors, RA enlargement, but not LA enlargement, was related to higher rates of clinical events. Among patients with LA enlargement, patients with RA enlargement had more clinical events than those without RA enlargement.

Atrial remodeling including atrial structural, architectural, contractile, or electrophysiological changes is crucial pathophysiologic findings in AF mechanisms (17, 19). While there are numerous studies regarding importance of LA structural and functional changes in AF, little is known about the factors which can attribute to RA enlargement and the prognostic value of RA remodeling in patients with AF. RA acts as a reservoir for systemic venous return when the tricuspid valve is closed. RA operates as a passive conduit in early diastole when the tricuspid valve is open and as an active conduit in late diastole during atrial contraction (19). In AF, decreased atrial contractile function and increased atrial compliance result in subsequent atrial remodeling and these changes will affect both LA and RA (20–22). RA size and its pressure can increase progressively in patients with AF, which can lead to an enlargement of the tricuspid annulus. At the same time, LV diastolic dysfunction and elevated LA pressure may increase the afterload of the right ventricle. These changes also may lead to a functional TR by participating in this vicious circle (23). Indeed, we observed that RA enlargement was related to LAVI, E/e’, and SPAP which can reflect LV diastolic dysfunction and elevated LA pressure, as well as TR grade in our data. We also showed RA enlargement was more significantly related to clinical events including HHF, stroke, systemic embolization or mortality than LA enlargement in patients with non-valvular AF. Although data about clinical utility of RA remodeling in patients with AF is limited, several literatures have reported the role of RA in patients with AF. Prior data have shown that the RA volume is superior to the LA volume in predicting AF recurrences at 6 months after direct current cardioversion (24). RA size is a significant predictor of arrhythmia recurrence after pulmonary vein isolation when compared with LA size (25, 26). Additionally, RA size is associated with stroke occurrence in patients with AF (27).

Rhythm control strategy including antiarrhythmic drugs or catheter ablation may effectively reduce the atrial remodeling by minimizing the burden of AF and it has shown to improve cardiovascular outcomes over usual care (28, 29). Rhythm control strategies may affect on the progression of atrial remodeling. In our study, the rhythm control strategy tended to yield better outcomes, however, it did not reach statistical significance probably due to the small number of patients with rhythm control. We additionally confirmed that RA size was consistently associated with clinical outcomes even after adjusting rhythm control strategy as an additional variable in the multivariate model.

The precise mechanisms of association of adverse clinical events in RAVI compared with LAVI are not clear. While RAVI was related to age, LV diastolic dysfunction, greater LV mass index, increased atrioventricular valvular regurgitation, and pulmonary hypertension, it was independently associated with adverse clinical events even after adjusting for clinical and echocardiographic parameters in our study. However, LA enlargement was not significantly related to clinical outcome in our data. Whereas LA enlargement is commonly used to assess LV diastolic dysfunction in patients with a variety of CV diseases, the LA can be enlarged regardless of filling pressure in cases of AF (18). On the other hand, there are some controversies with regards to LA enlargement as a risk factor of thromboembolic complications or the development of HF in patients with AF (6, 30, 31). To avoid the potential effect of factors other than AF on RA remodeling, we excluded the patients with chronic lung disease, intrinsic valvular disease, or other cardiomyopathies. Atrial enlargement is a part of atrial structural remodeling. Decreased atrial contractile function and increased atrial compliance in AF results in subsequent atrial remodeling and these changes will affect both LA and RA. Given the LA and RA volumes were more enlarged in patients with persistent or permanent AF than those with paroxysmal AF, both atrial anatomical remodeling might reflect the burden and the chronicity of AF (32). In addition, RA enlargement might reflect more advanced atrial remodeling rather than being a sensitive marker to hemodynamic loading compared with LA enlargement alone when considering that most patients with enlarged RAVI had an enlarged LAVI in our data. Therefore, RA remodeling which may reflect more chronicity of AF may contribute to worse clinical outcome through vicious pathophysiological cycle for HF and exposure to the persistent thromboembolic risk (33). Electrophysiologic remodeling followed by anatomical remodeling also may play a role in clinical outcome. Progression of atrial enlargement can increase the likelihood of ectopic or reentrant activity and it may produce a substrate for AF maintenance due to RA re-entrant activity, with an underlying substrate prominently involving RA fibrosis and conduction abnormalities (34).

In our study, only 3.5% of the patients had a history of a prior hospitalization for HF, indicating most of our population was in stage A or B according to 2022 ACC/AHA stages of HF (35). While we usually focus on LA enlargement or LV hypertrophy in structural changes in the pre-HF stage, we may need to pay more attention when RA enlargement develops. Further research would be necessary if earlier and more aggressive therapeutic intervention including catheter ablation or specific drugs can modify the relationship between RA remodeling and worse outcome in this population.

Several limitations of our study should be noted. First, our study was a retrospective cohort study and selective bias may occur. Second, we used 2D echocardiography to assess the LA and RA volumes. While 2D echocardiography is most commonly used to assess atrial size, it has inherent limitations because of the use of foreshortened views and asymmetric shapes of the atrium. In our study, we sought to avoid the use of foreshortened images to minimize measurement errors and excluded the patients if the images were not good enough to assess RA volume. Another study has shown that RA volume using single-plane Simpson’s method of disks from 2D echocardiography is comparable to those from MRI, whereas atrial volumes by 2D echocardiography are smaller than those by cardiac magnetic resonance (36, 37). However, good images would be essential in assessing RA volume accurately since it is common not to focus on the RA in clinical echocardiography. Indeed, we excluded 121 patients because of insufficient image quality including foreshortening or endocardial dropout to measure RA volume in our analysis. Third, the study population was relatively small and from a single referral tertiary center. Thus, our study population might be a skewed selected population, rather than representing the general population with AF. Indeed, our population showed more frequent incidence of adverse events than patients with non-valvular AF in the community.

## Conclusion

In patients with non-valvular AF, RA enlargement was more significantly related to adverse clinical events than LA enlargement, suggesting that RA size can be helpful in stratifying the risk in this population. Further studies are necessary to confirm the utility of RA size for AF in a larger population and whether specific therapies that reverse RA remodeling would improve clinical outcomes.

## Data availability statement

The raw data supporting the conclusions of this article will be made available by the authors, without undue reservation.

## Ethics statement

The studies involving human participants were reviewed and approved by Institutional Review Board (INHAUH2022-01-034-000). The patients/participants provided their written informed consent to participate in this study.

## Author contributions

K-YK, J-HJ, S-HC, Y-SB, SWK, S-DP, S-IW, D-HK, and S-HS: conception and design of the work, analysis and interpretation of data, writing of the original draft, and substantial revision of the manuscript. K-YK and J-HJ: data acquisition, analysis and interpretation of data, writing of the original draft, and substantial revision of the manuscript. S-HC, Y-SB, and SWK: data analysis and substantial revision of the manuscript. S-DP, S-IW, and D-HK: cross-checking of the analysis and substantial revision of the manuscript. All authors have contributed to critical revision of the manuscript and approved the submitted version.
